# Biomarker exploration of microRNA-203 as a promising substrate for predicting poor survival outcome in colorectal cancer

**DOI:** 10.1186/s12885-020-07512-x

**Published:** 2020-10-15

**Authors:** Qiliang Peng, Yi Shen, Peifeng Zhao, Shang Cai, Zhengyang Feng, Ming Cheng, Yongyou Wu, Yaqun Zhu

**Affiliations:** 1 Department of Radiotherapy & Oncology, The Second Affiliated Hospital of Soochow University, San Xiang Road No. 1055, Suzhou, 215004 Jiangsu China; 2 Institute of Radiotherapy & Oncology, Soochow University, Suzhou, China; 3 Department of Radiation Oncology, The Affiliated Suzhou Science & Technology Town Hospital of Nanjing Medical University, Suzhou, China; 4 Department of Oncology, The Second Affiliated Hospital of Soochow University, Suzhou, China; 5 Department of General Surgery, The Second Affiliated Hospital of Soochow University, Suzhou, China

**Keywords:** Colorectal cancer, microRNA-203, Biomarker, Prognosis

## Abstract

**Background:**

Increasing studies indicated that microRNA-203 (miR-203) may play an important part in the prognosis of CRC. Nevertheless, the prognostic and influential mechanism of miR-203 expression in CRC remains to be inconclusive. Accordingly, we conducted the current study to investigate the biomarker performance of miR-203 in CRC.

**Methods:**

In the present study, we conducted an evidence synthesis of the published literatures to identify the prognostic roles of miR-203 in patients with CRC. Moreover, several bioinformatics methods were applied for exploring the biomarker roles of miR-203.

**Results:**

It was demonstrated that elevated miR-203 expression was clearly related to worse overall survival (HR: 1.55, 95% CI: 1.07–2.24, *P* = 0.021) for CRC. The gene Ontology (GO) analysis indicated that miR-203 targets were primarily involved in a series of GO items closely associated with the molecular pathogenesis of CRC. The pathway analysis exhibited the potential signal pathways of miR-203 involved in CRC including pathways in cancer, wnt pathway, prolactin signaling pathway, proteoglycans in cancer, FoxO pathway, focal adhesion and Ras pathway. By constructing a protein-protein interaction (PPI) network of the targets of miR-203, ten crucial proteins and a significant network module were retrieved and found to serve important roles in the molecular pathogenesis of CRC.

**Conclusions:**

Our results indicated that miR-203 may function as a promising biomarker to monitor CRC survival outcomes and progression. Notably, large-scale prospective cohort studies and biological experiments are required to confirm our conclusions.

## Background

Colorectal cancer (CRC) is a significant health burden in both men and women, responsible for the third leading factor of cancer-related death [1]. In spite of the advances in treatment management and early screening, the survival outcome of CRC patients has not improved significantly [2]. It was found that the gene expression profiles of CRC at different sub-anatomical sites were different and their biological manifestations were also different, which may lead to different therapeutic effects and prognosis of the tumors with same TNM stage [3]. Thus, exploring a comprehensive biomarker for early predicting clinical outcomes CRC patients is essential for appropriate individual management.

MicroRNAs have been recognized as a subset of small and single-stranded noncoding RNAs which have emerged to be key regulators in various cellular processes containing differentiation, proliferation, apoptosis and stress response [4]. In human cancers, numerous studies have proved that microRNAs contribute to progression of angiogenesis, proliferation, migration, invasion, and survival of tumor cells [5]. Moreover, accumulative evidences have demonstrated microRNAs as stable molecular biomarkers in the diagnosis, prognosis and treatment response of some cancers [6].

Among these microRNAs, microRNA-203 (miR-203) is one of most commonly dramatically studied microRNAs in human cancers, with the location of the chromosome 14q32.33 [7]. Previous investigations revealed that the up-regulated miR-203 level was related to unsatisfactory clinical outcomes and high probability of carcinogenesis and recurrence in human malignancies including hepatocellular cancer, glioma, gastric cancer, breast cancer and CRC [8]. In CRC, miR-203 was reported to be associated with poor survival by regulating some genes in the pathogenesis of CRC [9]. Although miR-203 presented potential huge prognostic values in CRC patients, agreement has not been accomplished for the credibility of miR-203 for clinical application in CRC due to the existence of insignificant and opposing results. In addition, the potential mechanism in the occurrence and development of CRC is poorly evaluated.

Thus, we performed a comprehensive study of all eligible literatures to identify the relevance between miR-203 expression status and clinical outcomes of CRC patients. Moreover, an integrated bioinformatics analysis was also conducted for further understanding of prognostic value of miR-203 and its potential biomarker mechanism in CRC.

## Methods

### Literature retrieval strategy

The first evidence synthesis part was implemented following strictly with the guidelines of the PRISMA statement [10]. Relevant literatures were searched by systematically screening several electronic databases including PubMed, Web of Science and EMBASE until September 25, 2019. The following keywords were applied simultaneously: (microRNA-203 OR miR-203 OR miRNA-203), (colorectal OR rectum OR rectal OR colon) and (tumor OR neoplasm OR cancer OR malignancy)”. We also examined the reference lists for collecting potentially related studies.

Inclusion criteria were as follows: 1) investigated the association between miR-203 status and CRC overall survival (OS); 2) diagnosed CRC with surgical pathology; 3) provided the hazard ratio (HR) and 95% confidence intervals (CIs) directly or key information for estimating these parameters indirectly through Kaplan–Meier curves and original survival data.

Studies were excluded based on these exclusion criteria a: 1) lacked sufficient data; 2) letters, reviews, case reports or conference abstracts; 3) non-English studies.

### Data extraction and quality assessment

All included studies were independently assessed and extracted the data by two investigators (QP and YS). The following details of each article were recorded: i) first author’s last name and time of publication; ii) study location, patients number, distribution of age and gender and tumor stage; iii) miR-203 assay specimen and assessment method; iv) HRs and 95%CIs of elevated expression levels of miR-203 for OS. If the studies provided multiple types of survival data, we preferred the results of multivariate analysis as it considered confounding factors and has been demonstrate to be more accurate. If HRs were not offered by the original article, the statistical variables may be estimated from the Kaplan-Meier curves following the previously described method [11].

We applied Newcastle-Ottawa scale for evaluating the quality of each eligible article which was judged on three levels: selection, comparability, and outcome [12]. High quality required a NOS score ≥ 6.

### Statistical analysis for evidence synthesis

For the data pooling, HRs and the 95% CIs were calculated to elucidate the relevance between the expressing level of miR-203 and clinical survival. We selected Cochran’s Q test and Higgin’s I^2^ statistic to evaluate the heterogeneity of the studies [13]. When obvious heterogeneity was observed (*P* < 0.05), random-effects model would be taken to assess the combined effect size. If not, fixed-effects model may be selected [14]. We used several common methods including subgroup analysis, sensitive analysis, and meta-regression for seeking the potential sources of heterogeneity [15]. In order to test the publication bias, we adopted Begg’s test as well as Egger’s test [16]. *P*-value < 0.05 would be deemed as statistically significant.

### Identification of target genes of miR-203

The target genes of miR-203 were predicted using miRTarBase which is a database covers experimentally proved microRNA-target regulatory pairs based on reporter assays, western blot, or microarray experiments with up-regulated or down-regulated of microRNAs [17]. Updated in 2018, the database has become more powerful and comprehensive covering 422, 517 functional microRNA-target interactions according to 4076 microRNAs with 23, 054 regulated proteins retrieved from above 8500 literatures [18].

### Functional enrichment of miR-203

For determining the functions of miR-203, we implemented a comprehensive enrichment analysis with the targets of miR-203. Gene Ontology (GO) has been recognized as a commonly selected approach in the annotation of a large number of proteins while the KEGG pathway analysis could provide more valuable functional data about how molecules or genes are networked in different biological pathways [19, 20]. In the present study, KEGG and GO pathways were accomplished based on the Database for Annotation, Visualization and Integrated Discovery (DAVID) [21]. Subsequently, the cutoff value of prominent functional GO terms as well as pathway identification was established as *P* < 0.05.

### PPI network construction of miR-203

To gain the in-depth understanding of the biological functions of miR-203, we employed a PPI network analysis with the target genes of miR-203. We applied the Search Tool for the Retrieval of Interacting Genes (STRING) database for retrieving the predicted interactions among the regulated proteins of miR-203 [22]. A combined score of > 0.4 was deemed prominent and selected to establish the PPI network. Then, we visualized the network with the Cytoscape software for further analysis. Next, CytoNCA, a Cytoscape plugin, would be adopted to seek PPI network hub proteins, which offers several useful centrality measures, including betweenness centrality and closeness centrality and degree centrality [23]. Subsequently, the Cytoscape plug-in MCODE was applied to screen the remarkable modules of the set up PPI network. Finally, KEGG functional analysis of the crucial proteins and genes in the significant module was performed. *P*-value < 0 .05 would be reflected as statistically significant.

## Results

### Literature search and summary of included studies

**Fig. 1:**
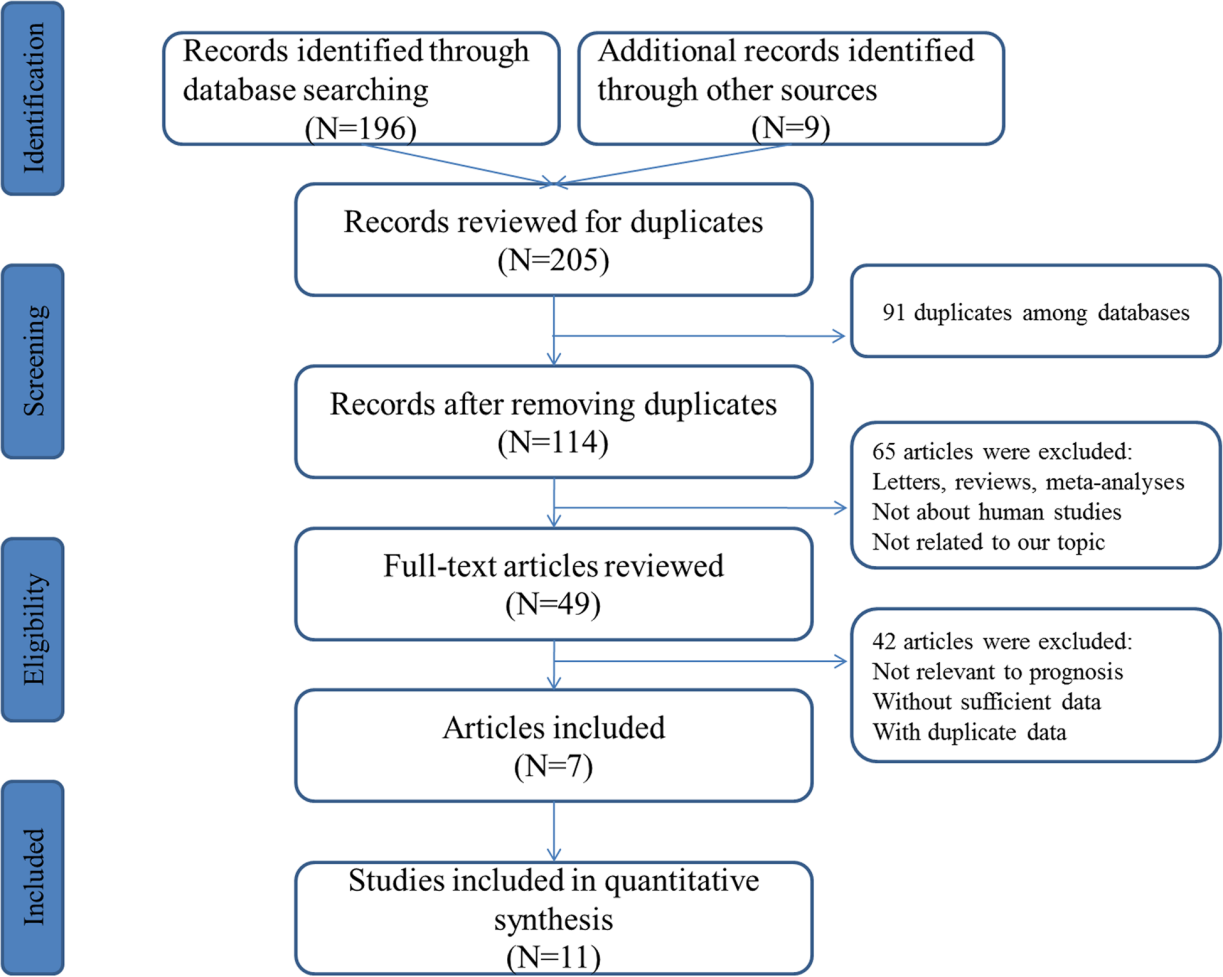
Flow diagram of literature search and selection

The workflow of research identification process was illustrated at Fig. 1. A total of 205 records associated with clinical outcomes of miR-203 and CRC were collected after primary screening. Then, publications which did not meet the eligibility criteria were excluded. Finally, 7 publications that represent 11 cohort studies were enrolled for the final evidence synthesis [24–30].

The principal features of each study were illustrated at Table 1. As a whole, 1452 cases from 7 papers containing 11 studies were included. The included papers were published ranging from 2008 to 2018. 7 studies were researched on Asians (3 from China and 4 from Japan) and 4 studies were about Americans. The studied specimens were categorized for either tissue (*n* = 6) or serum (*n* = 5). The expression of miR-203 was measured by quantitative real-time polymerase chain reaction (qRT-PCR). All the studies had scores from 6 to 9, which were categorized as high-quality studies.

**Table 1:** Main characteristics of the studies selected for the meta-analysis

First author	Year	Country	Ethnicity	M/F	N	Age	TNM stage	Sample souce	Methods	Endogenous control	Endpoints	Median follow-up time (months)	Hazard ratio
Schetter et al	2008	USA	Non-Asians	66/18	84	65	I-IV	Tissue	RT-PCR	U6	OS	68	3.10 (1.50–6.40)
Bovell et al	2013	USA	Non-Asians	170/175	345	65	I-IV	Tissue	RT-PCR	U6	OS	228	1.90 (1.05–3.42)
Tao et al	2014	China	Asians	47/43	90	70	I-IV	Tissue	RT-PCR	U6	OS	27	1.38 (0.77–2.48)
Deng et al. (Cohort 1)	2016	China	Asians	39/33	72	50	I-IV	Tissue	RT-PCR	U6	OS	84	0.62 (0.32–1.20)
Deng et al. (Cohort 2)	2016	China	Asians	39/33	72	50	I-IV	Serum	RT-PCR	U6	OS	84	0.36 (0.20–0.64)
Kingham et al. (Cohort 1)	2017	USA	Non-Asians	57/34	91	59	I-IV	Tissue	RT-PCR	miR-331	OS	192	1.92 (1.17–3.13)
Kingham et al. (Cohort 2)	2017	USA	Non-Asians	31/15	46	56	I-IV	Serum	RT-PCR	miR-331	OS	60	1.98 (1.14–3.42)
Takano et al	2017	Japan	Asians	147/93	240	NA	I-IV	Serum	RT-PCR	miR-16	OS	54	2.27 (1.31–4.09)
Hur et al. (Cohort 1-tissue)	2017	Japan	Asians	90/64	154	68	I-IV	Tissue	RT-PCR	Cel-miR-39	OS	72	1.56 (0.80–3.05)
Hur et al. (Cohort 1-serum)	2017	Japan	Asians	107/79	186	68	I-IV	Serum	RT-PCR	Cel-miR-39	OS	72	2.14 (1.09–4.21)
Hur et al. (Cohort 1-serum)	2017	Japan	Asians	87/57	144	68	I-IV	Serum	RT-PCR	Cel-miR-39	OS	72	2.35 (1.34–4.12)

Abbreviations: *F* female, *M* male, *N* number, *OS* overall survival

### Evidence synthesis and test of heterogeneity

**Fig. 2:**
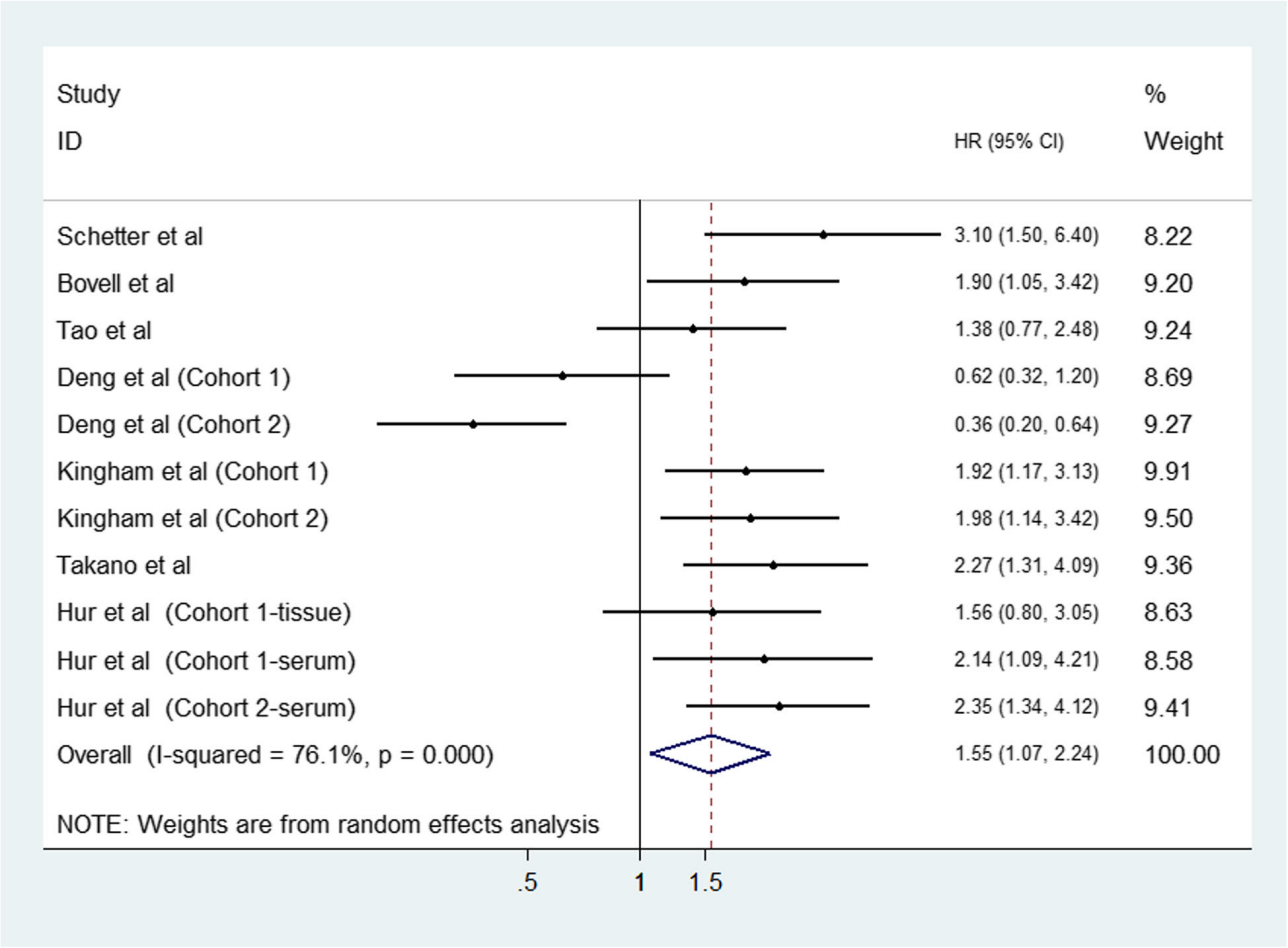
Forest plot for the association between miR-203 and overall survival of patients with colorectal cancer

Due to the obvious heterogeneity (I^2^ = 76.1%, *P* < 0.001), random-effects model was selected for measuring the association of miR-203 elevation and clinical outcome of CRC. The pooled analysis of the 11 studies revealed that elevated expression of miR-203 was demonstrated to be obviously correlated with shorter OS for CRC (HR: 1.55, 95% CI: 1.07–2.24, *P* = 0.021) (Fig. 2).

For explaining the heterogeneity in OS, subgroup analysis was further employed according to three subcategories containing sample source, sample size and the patient origin (Table 2). When stratified by ethnicity, high miR-203 status predicted worse survival in non-Asian patients (HR: 2.08; 95% CI: 1.56–2.77; *P* < 0.001), compared than Asian patients (HR: 1.28; 95% CI: 0.74–2.21; *P* = 0.384). The subgroup by specimens revealed the predictive role of miR-203 was especially more significant in tissue samples (HR: 1.57; 95% CI: 1.06–2.32; *P* = 0.023), but not in serum samples (HR: 1.52, 95% CI: 0.75–3.10, *P* = 0.250). Moreover, regarding the analysis of sample size subgroup, miR-203 exhibited a more prominent effect on predicting poor OS of CRC in large sample size (HR = 2.05; 95% CI: 1.07–2.24; *P* < 0.001) compared with small sample size (HR: 1.24; 95% CI: 0.66–2.31; *P* = 0.899).

**Table 2:** Results of subgroup and meta-regression analyses

Subgroup	Studies	HR (95%CI)	***P***-value	Heterogeneity (I^**2**^)	P_**heterogeneity**_	Meta-regression (***P***-value)
**Sample source**						*P =* 0.993
Serum	5	1.52 (0.75–3.10)	*P =* 0.250	86.5%	*P <* 0.001	
Tissue	6	1.57 (1.06–2.32)	*P =* 0.023	59.0%	*P =* 0.032	
**Sample size**						*P =* 0.078
Large(> 100)	5	2.05 (1.07–2.24)	*P <* 0.001	0.0%	*P <* 0.001	
Small(< 100)	6	1.24 (0.66–2.31)	*P =* 0.506	85.2%	*P =* 0.899	
**Ethnicity**						*P =* 0.080
Asian	7	1.28 (0.74–2.21)	*P =* 0.384	82.3%	*P <* 0.001	
Non-Asian	4	2.08 (1.56–2.77)	*P <* 0.001	0.0%	*P* = 0.709	

Furthermore, a meta-regression was also performed to seek the possible sources that may be responsible for heterogeneity. The results revealed that the following factors may potentially cause the heterogeneity but did not reach statistical significance: patient origins (*P* = 0.080), participant numbers (*P* = 0.078), and specimen sources (*P* = 0.993).

**Fig. 3:**
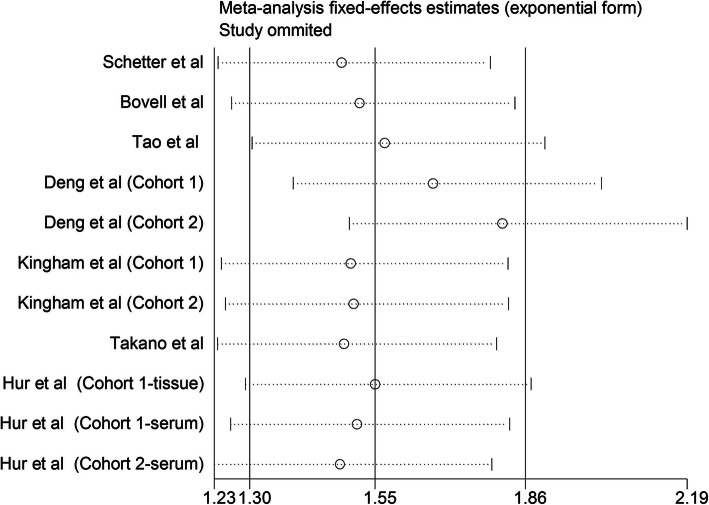
Consequence of sensitivity analysis

We then employed sensitivity analysis for investigating the impact of each individual study on the final results of OS by sequentially eliminating individual study at a time and calculating the combined HRs again. As shown in Fig. 3, no significant difference was observed after omitting any individual study, suggesting that our conclusions are stable.

### Evaluation of publication bias

**Fig. 4:**
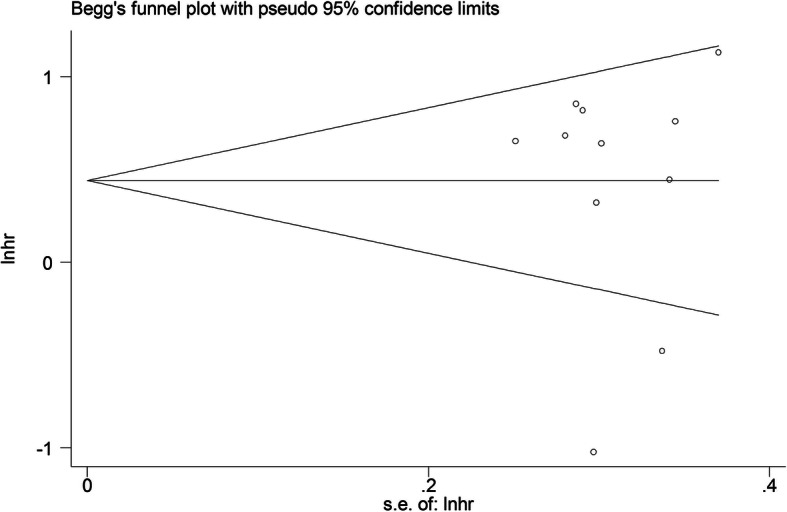
Funnel plot analysis of potential publication bias

We then employed Begg’s and Egger’s tests for detecting potential publication bias of the included articles. As displayed in Fig. 4, the funnel plot did not indicated any evidence of significant asymmetry; meanwhile, the *P*-value from the Egger test was 0.897 for OS, showing no existence of publication bias in the overall assessment of 11 enrolled studies.

### Functional enrichment analysis for miR-203

**Fig. 5:**
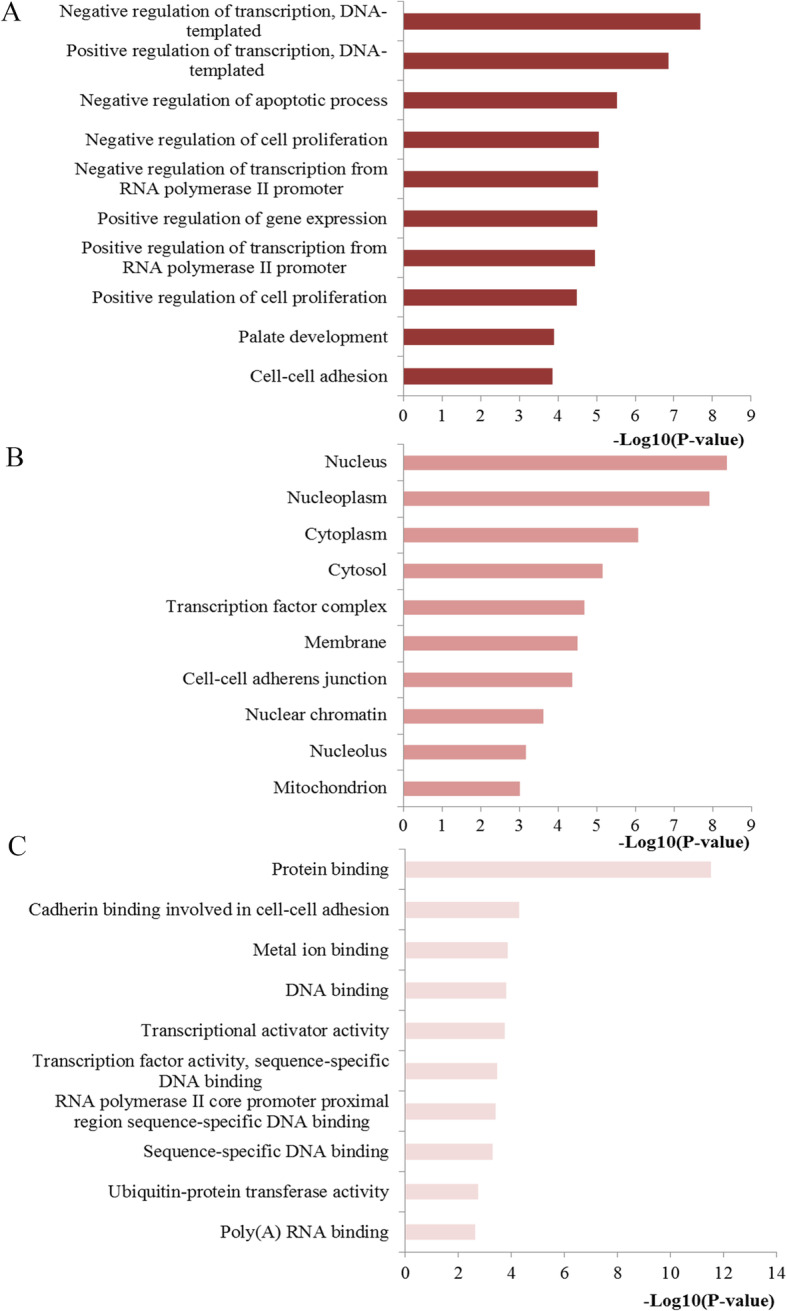
Top ten GO annotations of miR-203 target genes. **a** Biological processes; **b** cell component; **c** molecular function

To further understand why miR-203 can function to be a useful factor for predicting the clinical outcomes in CRC, we employed several bioinformatics methods to further explore the biological function of miR-203. GO and KEGG enrichment analysis are two common bioinformatics methods for delivering systematic, comprehensive bio-functional annotation information for a large amount of gene or protein lists. We collected a total of 574 target genes of miR-203 from miRTarBase and performed the GO and KEGG enrichment analysis. The top 10 enriched GO results are presented at Fig. 5. The results of the GO analysis revealed that in the enrichment analysis of biological process, the targets of miR-203 were primarily enriched in the regulation process including regulation of transcription, apoptotic process, cell proliferation and gene expression. In the enrichment analysis of cellular component, the targets of miR-203 were mainly enriched in nucleus, nucleoplasm, cytoplasm and cytosol. In the enrichment analysis of molecular function, the targets of miR-203 were significantly clustered in protein binding, cadherin binding, DNA binding and transcriptional activator activity.

**Fig. 6:**
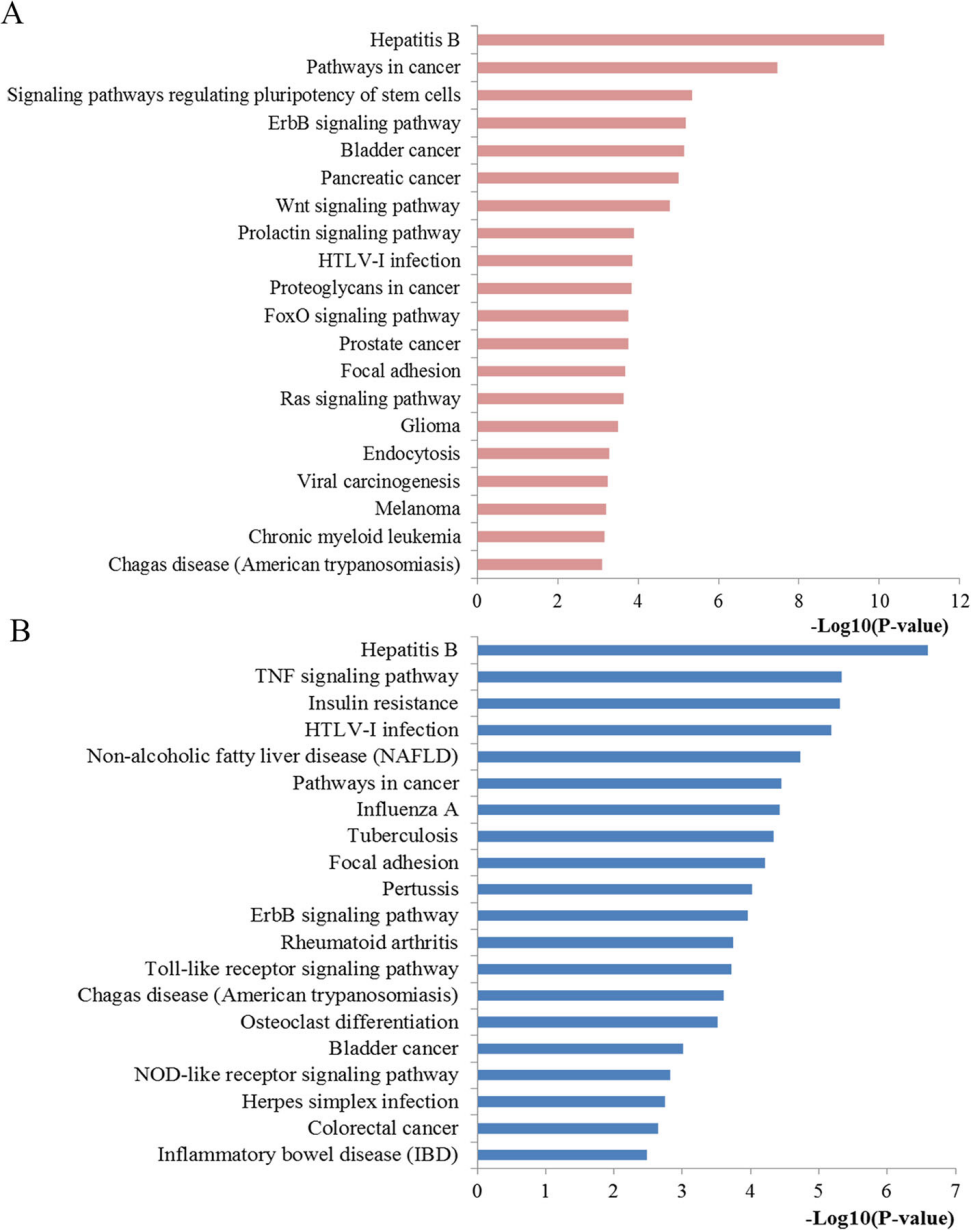
Pathway enrichment results. **a** Top 20 pathways enriched by all the target genes of miR-203; **b** Top 20 pathways enriched by the hub nodes of miR-203

**Fig. 7:**
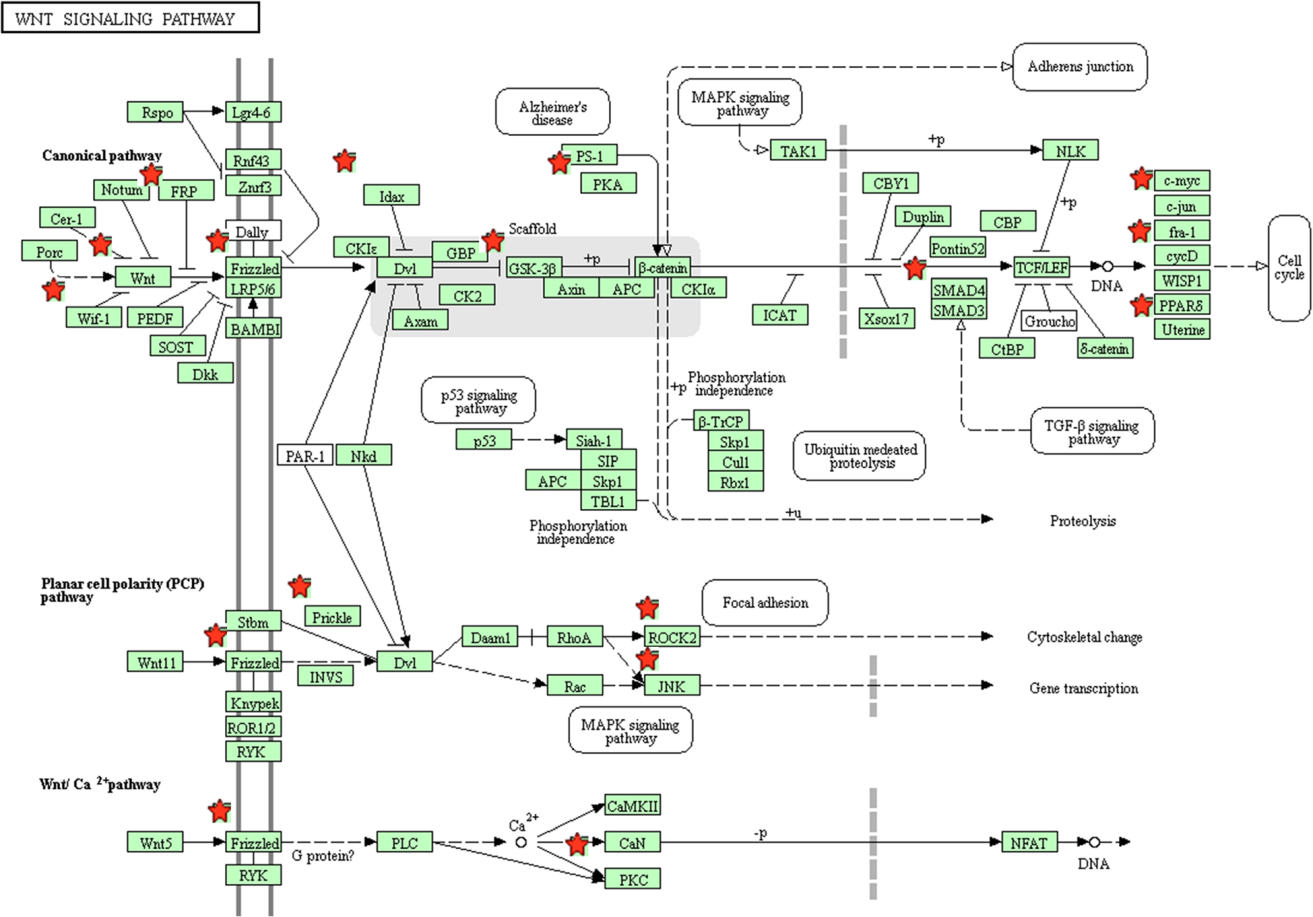
The Wnt signaling pathway enriched in KEGG. Objects with pentagrams are acting locus by mapped gene. The Wnt pathway was generated by KEGG database. KEGG, Kyoto Encyclopedia of Genes and Genomes

The top 20 prominently enriched KEGG signaling for miR-203 targets were also displayed at Fig. 6. The results revealed that miR-203 targets were particularly enriched in signaling including pathways in cancer, proteoglycans in cancer, focal adhesion, Wnt, prolactin, FoxO and Ras signaling pathway, as well as some types of cancer including glioma, bladder, pancreatic, and prostate cancer and. Selected as an example, the enriched Wnt signaling was also closely connected to p53, MAPK, cell cycle, adherens junction, and TGF-βsignaling (Fig. 7).

### PPI network construction and screening for key nodes

**Fig. 8:**
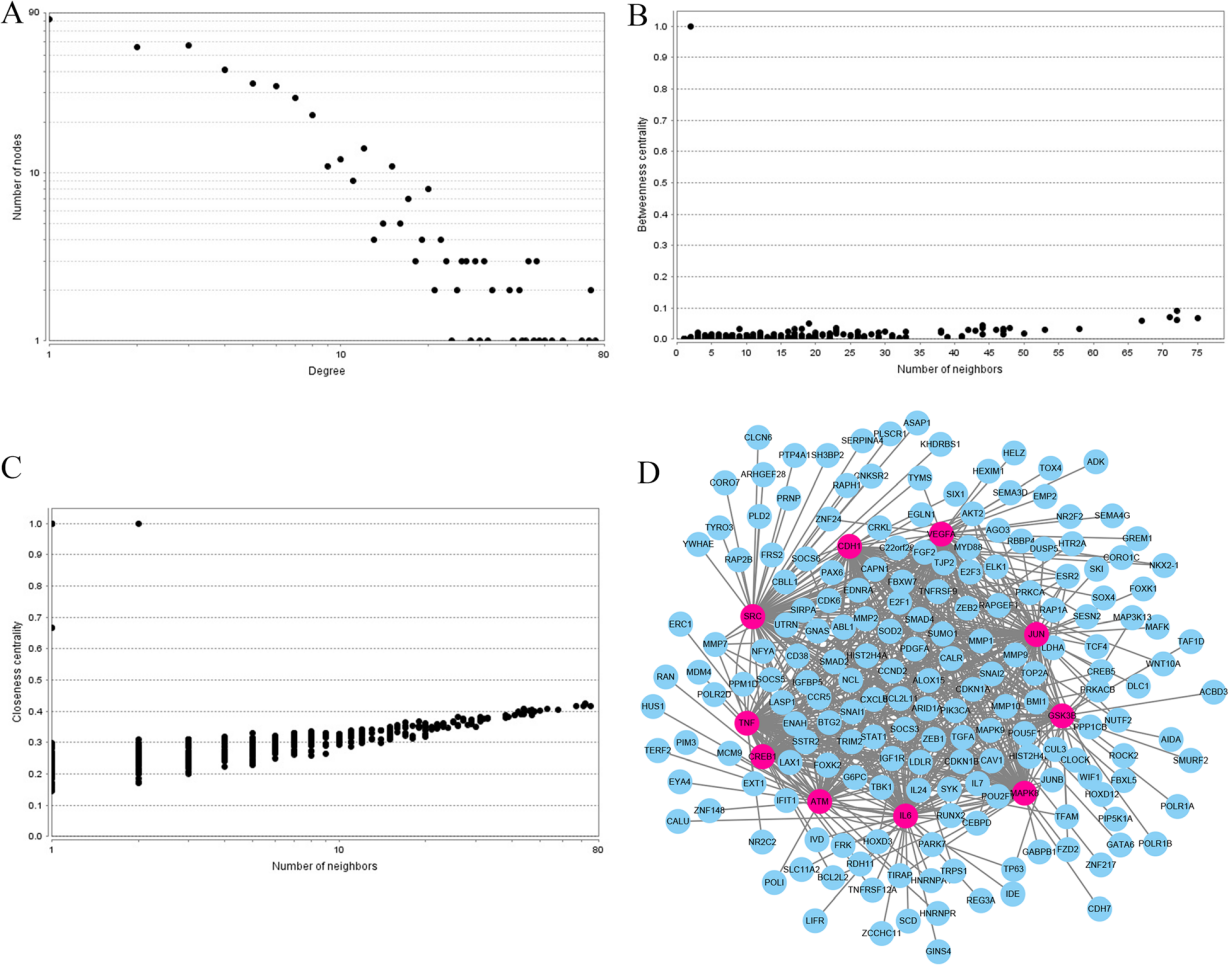
PPI network construction results. **a** Degree distributions of nodes; **b** Betweenness centrality distributions of nodes; **c** Closeness centrality distributions of nodes; **d** The sub-network reconstructed with the selected hub nodes and their first neighbor genes

The STRING online database was applied to analyze the 574 identified target genes of miR-203 and to set up a PPI network, which consisted of 498 nodes interacting with the 8.968 average numbers of neighbors. Subsequently, we employed Cytoscape tool for visualizing the network. To screen the most important target genes of miR-203, a centrality analysis through network establishment provide more information about the significance of the proteins within the network. In this study, the hub genes which were shared by all the three types of centralities were identified (Fig. 8). Following these criteria, the top ten hub genes were retrieved from the PPI network: *SRC*, *IL6*, *VEGFA*, *JUN*, *CDH1*, *GSK3B*, *ATM*, *CREB1*, *TNF* and *MAPK8*. A PPI network of the top 10 hub nodes was constructed through the STRING protein analysis and plotted at Fig. 8d. We explored the potential biological function through the KEGG pathway enrichment analysis. According to the results, these hub genes were predominantly involved in TNF, Toll-like receptor, NOD-like receptor, colorectal cancer and pathways in cancer signaling.

### Module analysis of PPI network

**Fig. 9:**
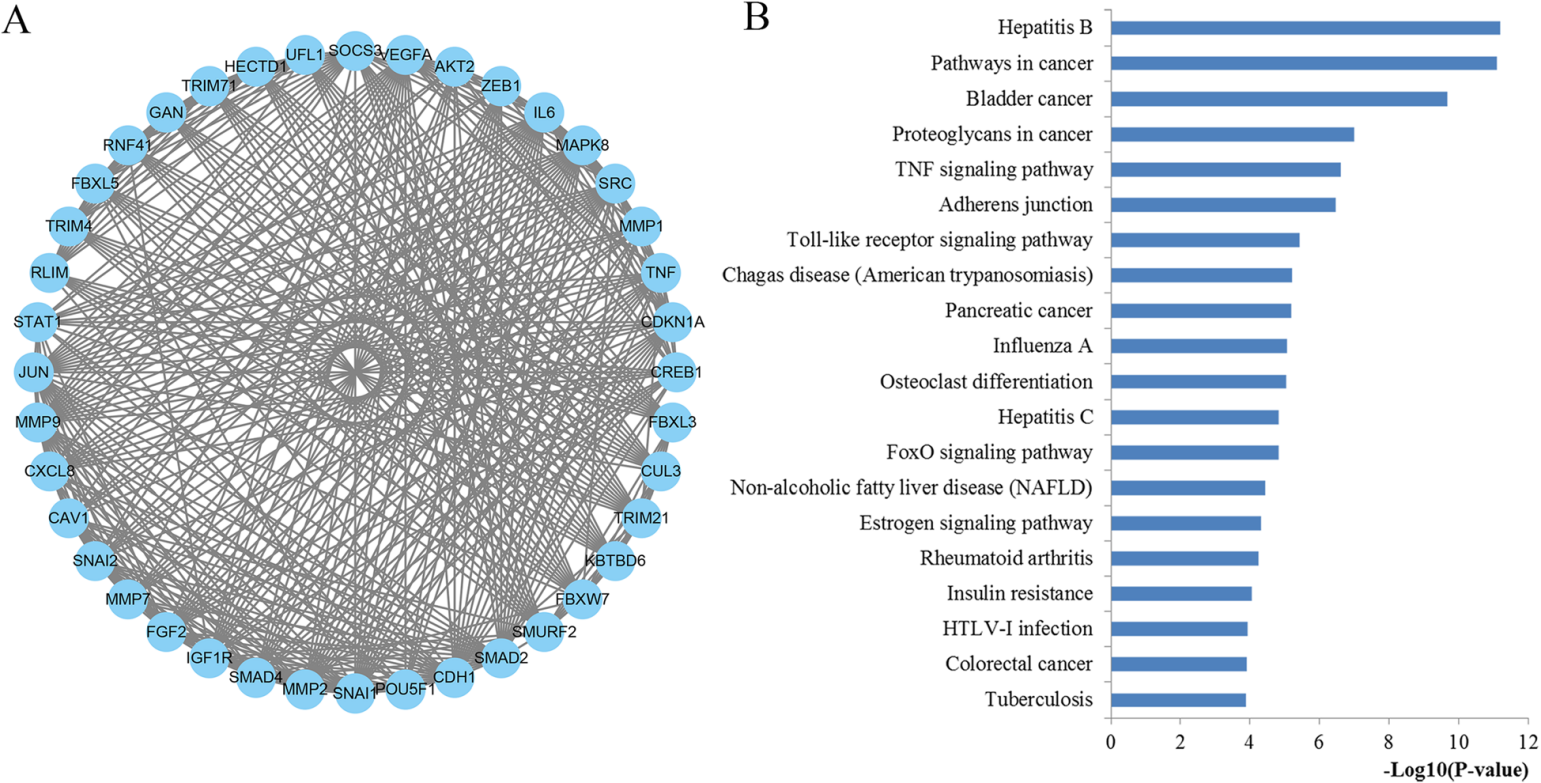
Module analysis results of the PPI network. **a** The most significant module in the PPI network; **b** Pathways enriched by all the nodes involved in the identified module

Module in the network may affect the stability and overall function of the network. In this study, the Cytoscape plug-in MCODE was applied to screen the significant modules of the constructed PPI network. A total of 15 modules were identified by MCODE plug-in. According to the MCODE score (19.64), the module consisted of 40 nodes and 383 edges were identified to be the most significant. The nodes involved in the screened module were conducted function and pathway enrichment analysis, which were significantly involved in the pathways in cancer, proteoglycans in cancer, adherens junction, TNF, toll-like receptor, FoxO and colorectal cancer signaling as well as some other cancers including bladder cancer and pancreatic cancer (Fig. 9).

## Discussion

CRC is still a deadly threat in human health because of tumor metastasis and relapse contributing to locally advanced stages and unsatisfactory survival outcome. In recent decades, the exploration of useful biomarkers for clinical application remains to be the focus of research due to the complexity of CRC. Emerging studies have found that aberrant expression of microRNAs associated with cancers, and some specific members have been identified with important impact during cell growth, migration, invasion and cancer progression and thus could be novel potential tools for predicting cancer initiation and survival outcome. As one of the most frequently mentioned members in CRC, miR-203 has been identified to play a significant part in tumorigenesis and CRC occurrence and development by adjusting the expression level of potential targets. However, it is controversial regarding the prognostic performance of miR-203 for CRC due to the inconsistent results from different studies. For example, recently, Schetter et al. [24] found higher level of miR-203 might be associated with worse clinical outcome. On the other hand, Deng et al. [27] analyzed the value of serum miR-203 and yielded a conclusion that circulating higher miR-203 levels had a better overall survival in CRC. Hence, in order to evaluate the prognostic role of miR-203 status in CRC and uncover the potential mechanism of miR-203 involved in CRC, we systematically reviewed the published studies and performed this study for addressing the relevance between miR-203 and survival outcome of CRC patients and explored the potential mechanisms.

The results indicated that miR-203 could be applied to be a useful biomarker for prognosis prediction, as high expression miR-203 was associated with worse overall survival in CRC (HR: 1.55, 95% CI: 1.07–2.24) with a *P*-value of 0.021. Notably, obvious heterogeneity was found across these studies, with a *P*-value < 0.001. Stratified analyses indicated that ethnicity, sample size and sample source might be the major factors of heterogeneity. In detail, the predictive value of miR-203 was more significant in non-Asians, large sample size and tissue samples than in Asians, small sample size and serum samples, respectively, which provided new ideas of designs of clinical trials or scientific research for promoting miR-203 into clinical application. Correspondingly, more studies on these points are required for further investigation.

The above clinical data synthesis part has suggested the usefulness of miR-203 expression as a promising biomarker in predicting the survival outcomes for CRC. However, the mechanisms how miR-203 affected the initiation and progression of CRC was not fully understood. The molecular pathogenesis of CRC mainly comes from multistep, multifactor, and polygenic effects and involves changes in various oncogenes and tumor associated microRNAs. Thus we applied several bioinformatics methods for exploring the biological function of miR-203 at the functional level. Since microRNAs play the biological role by regulating downstream genes, we first conducted a functional enrichment analysis including GO and KEGG analysis with the regulated genes of miR-203. The GO analysis demonstrated that the genes regulated by miR-203 were primarily involved in regulation process for the biological process level, mainly enriched in basic cellular component for the cellular component level and significantly clustered in binding function and transcriptional activator activity for the molecular function level. KEGG pathway analysis indicated that some of the signaling pathways regulated by the target genes of miR-203 were involved in some important pathways. The majority of these signaling pathways have been demonstrated significantly related to the occurrence and development for CRC. For example, pathways in cancer may be the most crucial pathway due to the coverage of a series of pivotal signaling, for example the p53, TGF-β, Wnt and MAPK signal pathway, which have dominant impact on almost all the aspects of the tumorigenesis and progression of cancers including CRC. Accumulating evidence suggests that Wnt pathway is important during the pathogenesis of various types of cancers including CRC through influencing cellular proliferation, differentiation, and motility [31]. The abnormal Wnt signaling may lead to carcinogenesis and targeting this pathway may represent a potential strategy for cancer therapy [32]. Proteoglycans in cancer, identified as critical constituents of the extracellular matrix, take significant part in the structural organization of the extracellular matrix and cell signaling which may lead to the control of extensive normal and pathological processes [33]. Moreover, increasing evidence has confirmed that FoxO pathway has a crucial influence on the regulation of cellular homeostasis from development, cell signaling, and tumorigenesis to cell metabolism [34, 35]. Focal adhesion, a cytoplasmic receptor tyrosine kinase, plays a prominent role in controlling essential cellular activities containing cell growth, proliferation, survival and migration [36]. In addition, Ras signaling pathway, a well-studied pathway for CRC, the dysregulation of which plays a pivotal part during the progression of CRC and targeting this signaling may provide a potential therapeutic target in the therapy for CRC [37]. The aforementioned results revealed a possible function of miR-203 and the related pathways during CRC pathogenesis.

As microRNA regulates multiple target genes, and each target gene has close connections with others. So we carried out a PPI analysis of genes regulated by miR-203 to explore more valuable information about miR-203. Next, based on the PPI network construction and analysis, top ten crucial genes were obtained. The crucial genes were demonstrated closely associated with some important pathways, which had pivotal impact on the occurrence and progression according to literature exploration. The most important module of the network map was further identified. The KEGG pathway analysis revealed that proteins of the prominent module were significantly enriched into a series of vital pathways. Most of the pathways have been confirmed by previous literatures. Here, we have to mention some more important pathways. It is worth noting that TNF, as an extraordinarily pleiotropic cytokine, is highly associated with maintenance and homeostasis of the immune system, inflammation as well as host defense [38]. Adherens junction plays a central role in CRC carcinogenesis and targeting it may promote CRC progression [39]. Moreover, accumulating preclinical evidence indicates that Toll-like receptor pathway participates in stimulating innate and adaptive immune responses and has a pivotal part during diseases induced by inflammation such as CRC [40]. Recent reports indicated that targeting this pathway may be beneficial for CRC treatment [41]. In addition, it is well established that NOD-like receptor plays crucial and complex roles in the homeostasis of the immune system. The abnormal activation of the signaling could cause carcinogenesis and tumor invasion [42]. The colorectal cancer directly demonstrated that the hub genes were significantly related to the initiation and progression for CRC [43]. The PPI analysis results are reliably consistent with the present studies. The identified genes, pathways and modules revealed the potential mechanisms of miR-203 involved within the pathological processes of CRC and provided novel insights for the treatment strategy of CRC.

Recently, some studies on the tumor suppressive functions of miR-203 have been investigated. For example, recently, You et al. [44] reported that miR-203 restrains epithelial-mesenchymal transition (EMT), invasion and migration of papillary thyroid cancer by down-regulating AKT3. On the other hand, Lai et al. [45] revealed that miR-203 could diminish the stemness of human colon cancer cells through suppressing GATA6 expression. Moreover, growing evidence from recent studies has revealed that miR-203-regulated posttranscriptional deregulation of CPEB4 may promote the progression of CRC and directly targeting CPEB4 by miR-203 might be a novel strategy in CRC treatment [46]. These studies together with the findings from our bioinformatics analysis may provide help for further understanding the tumor suppressive functions of miR-203 involved in CRC.

To the most of our knowledge, our study may be the most comprehensive study about miR-203 as we not merely quantifies the biomarker performance of miR-203 for predicting the prognosis of CRC by meta-analysis, but also qualitatively explores the potential function and mechanism of miR-203 through integrative bioinformatics analysis. On the whole, our quantitative results strongly agreed with the present mainstream viewpoint that an undesirable impact of miR-203 high expression was related to worse clinical outcome. Moreover, there are still numerous valuable implications from our interesting results for future clinical and scientific research. In addition, although we have not conducted experiment, most of the enriched modules, pathways or networks were successfully confirmed by recent experimental literatures.

However, several limitations exist in the present study. Firstly, the number of studies was relatively small. More clinical studies should be conducted to extend and validate the conclusions in the future. Secondly, we collected and pooled data from public studies rather than individual case data, so subgroup and regression analyses focusing on some important variables containing age, sex and stage of cancer were restricted due to the insufficient data. Thirdly, we only enrolled patients from Asians and Caucasians; so further studies assessed the biomarker roles of miR-203 about more ethnicities are required as the study population are not comprehensive. Besides, language bias may exist as only English studies were enrolled for evidence synthesis which may published more positive results. Due to the fact that all studies that are used for the data pooling have patients of various TNM stage (from stage I to stage IV), there is considerable heterogeneity in survival. Without patient-level data, it would be very difficult to tell whether miR-203 is actually useful as a prognostic factor. Finally, though we have validated the relevance of miR-203 status with the prognosis of CRC and proved the accuracy of our predictive results through an integrated bioinformatics analysis, well-designed biological experiments are still required for future confirmation as the initiation and development of CRC is complex, with multiple cumulative genetic changes.

## Conclusions

It was found that up-regulated miR-203 level may be a risk factor for CRC prognosis revealing a poor OS. Some vital genes, pathways and modules were identified through an integrated bioinformatics analysis, indicating the potential mechanisms of miR-203 during the initiation and progression of CRC. More large-scale and multicenter prospective clinical studies and biological experiments should be performed before the practical implementation for miR-203 regarding the survival prediction of CRC patients.

## Data Availability

Data is available from the corresponding author upon reasonable request.

## Abbreviations

CRC: Colorectal cancer
HR: Hazard ratio
CI: Confidence interval
GO: Gene ontology
KEGG: Kyoto Encyclopedia of Genes and Genomes
DAVID: Database for Annotation, Visualization, and Integrated Discovery
BP: Biological process
CC: Cellular component
MF: Molecular function
